# A case of pembrolizumab-induced bullous pemphigoid treated with dupilumab

**DOI:** 10.1093/skinhd/vzae023

**Published:** 2025-02-14

**Authors:** Agnese Rossi, Donatella Brancorsini, Helena Gioacchini, Anna Campanati

**Affiliations:** Dermatology Department, Polytechnic University of Marche, Ancona, Marche, Italy; Department of Biomedical Sciences, Polytechnic University of Marche, Ancona, Italy; Dermatology Department, Polytechnic University of Marche, Ancona, Marche, Italy; Dermatology Department, Polytechnic University of Marche, Ancona, Marche, Italy

## Abstract

Immune checkpoint inhibitors are a class of drugs used in cancer treatment that promote the immune system’s response by blocking the inhibitor signals from tumour cells, such as programmed cell death protein 1/programmed death ligand 1 and cytotoxic T-lymphocyte associated protein 4. Despite their clinical benefit, these monoclonal antibodies unspecifically activate the immune system and can lead to the development of ‘immune-related adverse events’. Cutaneous toxicities are the most frequent immune-related adverse events, reported in approximately 30–50% of patients treated with immunotherapy; the most common dermatological toxicities are represented by rash, vitiligo, pruritus and lichenoid reactions. Usually, these reactions are mild and it is not necessary to suspend immunotherapy. Potentially life-threatening skin toxicities, such as immunobullous eruption, are rare and may appear in approximately 1% of patients. In this report we describe a case of bullous pemphigoid, the most frequent bullous disease, that developed after treatment with pembrolizumab for a metastatic melanoma. The diagnosis, first suspected by the referring clinic, was confirmed by performing serology and biopsy with direct immunofluorescence. The patient was first treated with high doses of systemic corticosteroids, without suspending the immunotherapy treatment. Subsequently, due to the continuous relapses, we decided to suspend pembrolizumab and systemic corticosteroid and to begin off-label treatment with dupilumab. The following case gives cause for reflection about the management of a drug-induced disease in an immunocompromised patient, while exploring the therapeutic options.

## Teaser

A clinical case showing the efficacy of dupilumab on pembrolizumab-induced bullous pemphigoid.

**What is already known about this topic?**

- Bullous pemphigoid is a rare but potentially severe adverse event during immunotherapy; its treatment may be challenging, due to poor responsiveness to conventional treatment.
- Recently, new and effective treatments, such as monoclonal antibodies, have been proposed.

**What does this study add?**

- This case is a reflection on the management of a drug-induced disease in an immunocompromised patient, which underlines the efficacy and safety of treatment with dupilumab.

##  

In November 2020 a 67-year-old man was referred to our hospital for the development of an itchy bullous eruption. Physical examination showed tense blisters and erosions overlying erythematous plaques on the arms, especially axillae, face, back, chest, abdomen, legs and back of the feet. He had no mucosal involvement.

Ten months before the onset of the eruption, he had begun immunotherapy with pembrolizumab (200 mg every 3 weeks) for an axillary and laterocervical lymph node recurrence of a stage IIIA BRAF wildtype melanoma, according to the eighth edition of the American Joint Committee on Cancer’s Staging Manual. The tumour was localized on the back and had been removed 7 years earlier, in conjunction with dissection of axillary and laterocervical lymph nodes.

**Figure 1:**
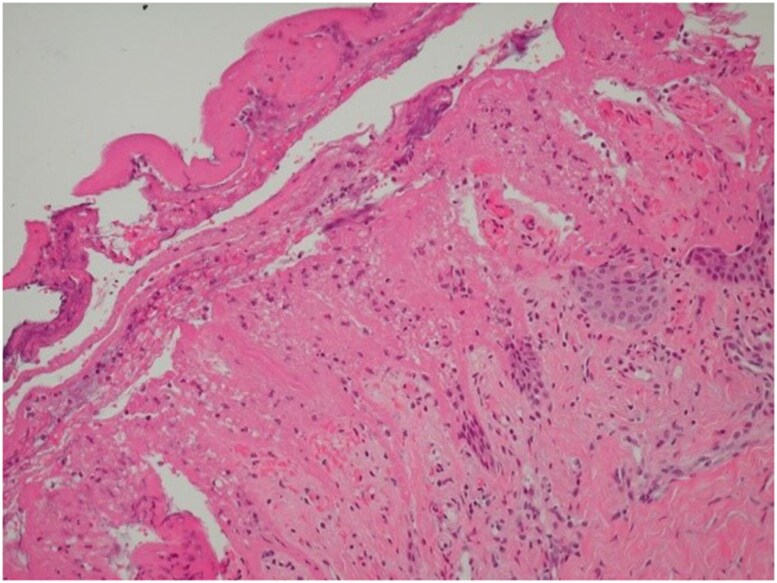
Histopathology. Skin biopsy from the chest, showing subepidermal bulla along with dermal inflammatory cell infiltration. Direct immunofluorescence demonstrates deposition of both IgG and complement C3 at the dermoepidermal junction.

Skin biopsy was performed and showed subepidermal blisters along with dermal eosinophils infiltration, whereas direct immunofluorescence (DIF) revealed deposits of IgG and complement C3 along the dermoepidermal junction (Figure 1). Blood tests revealed an increased numbers of eosinophils (0.73 × 10^9^ cells L^–1^; range 0.01–0.50) and neutrophils (13.09 × 10^9^ cells L^–1^; range 40–75), with high levels of anti-BP180 (69 U mL^–1^; cutoff <19.99) and IgE (150 U mL^–1^; cutoff <130) autoantibodies. The detection of anti-BP230 autoantibodies was negative. All of these elements, in conjunction with the timeline of start of pembrolizumab, supported a diagnosis of pembrolizumab-­induced bullous pemphigoid (BP).

**Figure 2:**
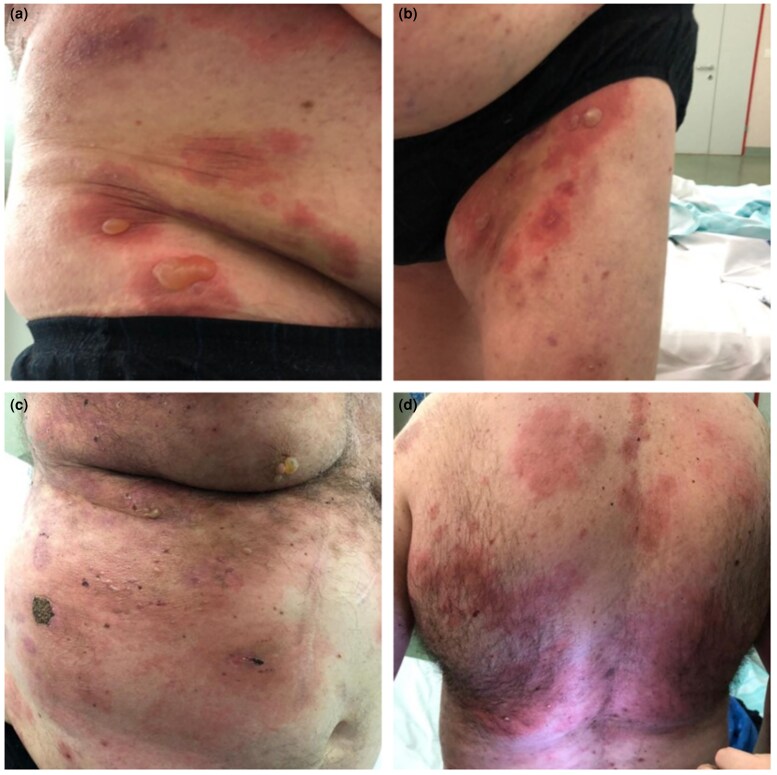
Clinical images. (a, b) Tense blisters on erythematous plaques localized on abdomen and upper thighs. This reaction was classified as being of grade 2 severity. (c, d) Extensive bullous eruption with diffuse erythema and eroded blisters. All body areas were affected, determining a grade 3 immune-related adverse event.

This reaction was classified as being of severity grade 2 (Figure 2a, b), due to the involvement of 10–30% body surface area (BSA), and the patient was treated as an outpatient with oral prednisone 10 mg daily and with topical clobetasol propionate twice daily, without interrupting immunotherapy. The bullous rash was partially controlled with this treatment, but tended to recur at tapering of systemic steroid, with the need for repeated high-dose steroid cycles.

In November 2021, due to the development of steroid-induced diabetes mellitus and Cushing disease, prednisone was lowered to the minimum effective dose of 5 mg daily and treatment with mycophenolate mofetil 2 g daily was begun. The patient continued to apply clobetasol propionate twice daily. We preferred mycophenolate mofetil to azathioprine because of its better safety profile–­azathioprine is associated with gastrointestinal intolerance, bone marrow suppression and infections. In contrast, treatment with mycophenolate mofetil is usually well tolerated; gastrointestinal distress is the most common side-effect.

After only 1 month, in December 2021, the patient developed a grade 3 bullous reaction (Figure 2c, d), with the involvement of 30% BSA, requiring hospitalization and discontinuation of mycophenolate mofetil and pembrolizumab. During hospitalization, the patient received combination treatment with betamethasone 5.5 mg daily intravenously and immunoglobulins 0.5 mg kg^–1^ for 4 days, with temporary partial benefit and new relapse at steroid tapering, with further rehospitalization the following month.

**Figure 3:**
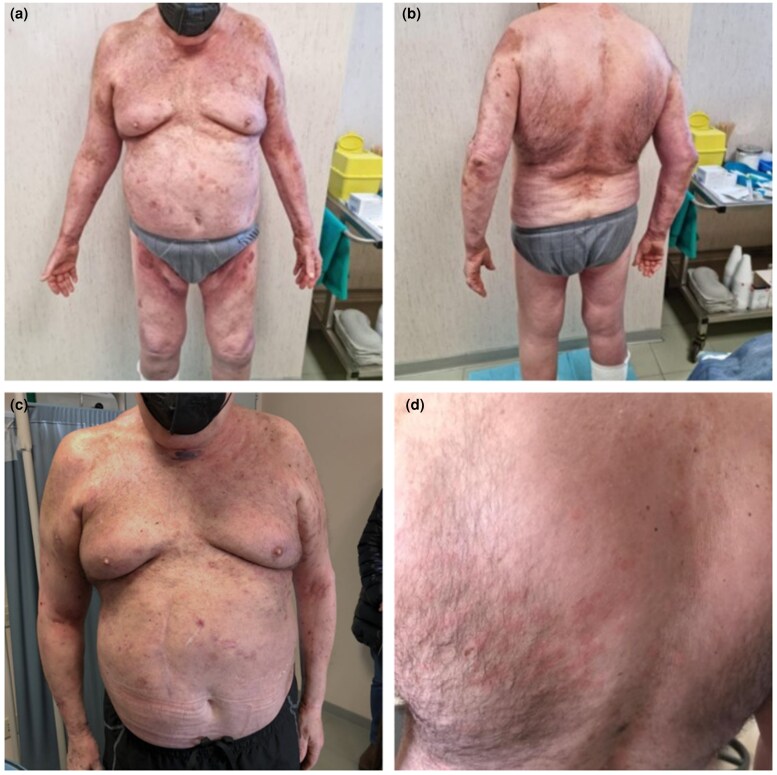
Clinical images. (a, b) Evaluation of the patient after 8 weeks of treatment: a noticeable improvement was seen, with a reduction in erythema and bullous involvement. (c, d) Evaluation of the patient after 16 months: near resolution of the disease.

The difficult management of skin toxicity resistant to conventional systemic treatments and the inability to resort to prolonged use of the immunosuppressive drugs commonly used in BP, led us to try dupilumab. In January 2022 the patient began treatment with dupilumab 300 mg via subcutaneous injection administered every other week, after a 600-mg loading dose, in conjunction with betamethasone tapering beginning at 5.5 mg daily. After 8 weeks of treatment, the patient showed an improvement in the cutaneous disease, with a reduction in itch and new bullae development. After an additional 2 months, he presented only sporadic erythematous areas, as an outcome of previous lesions (Figure 3). During treatment the patient did not experience any side-effects.

## Discussion

We describe a patient with BP as an immune-related adverse event, unresponsive to conventional treatment and successfully treated with dupilumab.

The precise pathogenesis underlying the development of pembrolizumab-induced BP has not been completely identified. Some studies suggest that it could be linked to the presence of homologous antigens between tumour cells and heathy cells. In fact, BP180, the main target of BP’s antibodies, has been found on cancers cells, such as non-small cell lung cancer or melanoma cells, and on skin basement membrane. It is possible that BP arises as a result of overactive T cells, uninhibited by immunotherapy, targeting BP180 on tumour cells and basement membrane.^1–4^ According to some studies, these adverse cutaneous reactions have a positive value, because they are associated with a higher rate of treatment success and longer progression-free disease survival. However, the difference in outcomes of patients with BP is still unclear and more studies are need to confirmed this aspect.^1,2^

Immunotherapy-related BP has a clinical presentation similar to classical BP, with the arise of bullous lesions usually within 6–10 months of initiating programmed cell death protein 1 (PD-1)/programmed death ligand 1 (PD-L1) inhibitors, as happened in our patient; there is also a small number of patients who do not develop bullae for 1–1.5 years following initiation of therapy.^3^ The diagnosis in our patient was confirmed by performing a biopsy with DIF and an enzyme-linked immunosorbent assay, which were positive for anti-BP180 antibodies; anti-BP230 antibodies were untraceable.^4^ These results are in agreement with the literature, which underlines how anti-BP180 antibodies are present a significantly higher positive rate than anti-BP230 antibodies in pembrolizumab-induced BP.^2^

Regarding treatment, the literature shows that pembrolizumab-induced BP frequently requires discontinuation of PD-1/PD-L1 inhibitors; despite this, some patients experience prolonged courses of BP with intermittent blister recurrence occurring 3–12 months following cessation of checkpoint inhibitors.^5,6^ In most cases, BP is treated successfully by a combination of oral and topical steroids, but in other cases conventional immunosuppressive drugs (methotrexate, azathioprine, mycophenolate mofetil, cyclophosphamide or ciclosporin) were needed. For the most severe cases of BP refractory to corticosteroid and conventional immunosuppressive drugs, many studies have reported the use of intravenous immunoglobulin, plasma exchange, immunoadsorption and off-label biologics such as dupilumab, rituximab and omalizumab. In our case, we preferred dupilumab over rituximab and omalizumab because studies have shown that the incidence of adverse events is more common with omalizumab and rituximab than with dupilumab.^7–9^

Dupilumab is a fully humanized IgG4 monoclonal antibody that targets the alpha subunit of the interleukin (IL)-4 receptor and consequently inhibits IL-4 and IL-13 signal transduction. These cytokines, produced by a subpopulation of CD4 T cells, stimulate type 2 immunity, which is characterized by high levels of IgE antibodies and eosinophilia. Several studies have underlined a prominent type 2 inflammatory response in BP.^9–11^

Dupilumab is described as a more effective and safe treatment for BP compared with conventional treatment with immunosuppressive drugs. Moreover, the long-term use of immunosuppressive drugs is not recommended in patients with cancer, because it could compromise antitumour immunity.^10–14^

The interruption of immunotherapy in the case of immune-related adverse events is controversial and, to date, there is no validated evidence from literature to indicate the best approach to manage these patients. Usually, immunotherapy is continued in patient with a mild/moderate BP (toxicity grades 1–2), especially if it can be controlled with topical or systemic corticosteroids; however, in case of severe reaction (grade 3–4), they must be immediately suspended.^2–15^

In our patient it was essential to use a systemic treatment alternative to corticosteroids, due to the refractoriness and severity of the disease, which seemed unlikely to undergo spontaneous clinical remission despite cessation of the drug.

In conclusion, patients who receive immunotherapy may develop cutaneous adverse events, including BP. Its treatment may be challenging and clinicians should consider the use of new immunomodulatory drugs, such as dupilumab.

## Data Availability

The data underlying this article are available in the article.
